# Electroconvulsive therapy for treating patients with agitation and related behavioral disorders due to dementia: a systematic review

**DOI:** 10.1590/1980-5764-DN-2023-0007

**Published:** 2023-07-24

**Authors:** Florindo Stella, Márcia Radanovic, José Gallucci-Neto, Orestes Vicente Forlenza

**Affiliations:** 1 Universidade de São Paulo, Faculdade de Medicina, Instituto de Psiquiatria, LIM-27, São Paulo SP, Brazil.; 2 Universidade Estadual Paulista, Instituto de Biociências, Rio Claro SP, Brazil.; 3 Universidade de São Paulo, Faculdade de Medicina, Instituto de Psiquiatria, Serviço de ECT, São Paulo SP, Brazil.

**Keywords:** Aggression, Dementia, Electroconvulsive Therapy, Behavioral and Psychological Symptoms of Dementia, Agressão, Demência, Eletroconvulsoterapia, Sintomas Psicológicos e Comportametais da Demência

## Abstract

Behavioral disturbances are clinically relevant in patients with dementia, and pharmacological regimens to mitigate these symptoms have provided limited results. Proven to be effective in several psychiatric conditions, electroconvulsive therapy is a potentially beneficial strategy for treating severe agitation due to dementia.

**Objective:**

This review aimed to examine the publications on the efficacy, safety and tolerability of electroconvulsive therapy in treating patients with agitation due to dementia.

**Methods:**

We performed a systematic analysis on the electroconvulsive therapy to treat patients with dementia and coexisting severe agitation. Articles were classified according to the level of evidence based on methodological design. Patients received an acute course of electroconvulsive therapy, often followed by maintenance intervention.

**Results:**

We selected 19 studies (156 patients; 64.1% women; 51–98 years old), which met the inclusion criteria: one case-control study by chart analysis (level of evidence 2); one open-label study (level of evidence 3); three historical/retrospective chart analyses (level of evidence 4); and 14 case series/reports (level of evidence 5). No randomized, sham-controlled clinical trials (level of evidence 1) were identified, which represents the main methodological weakness. Some patients had postictal delirium, cardiovascular decompensation and cognitive changes, lasting for a short time.

**Conclusions:**

Overall, patients achieved significant improvement in agitation. However, the main finding of the present review was the absence of methodological design based on randomized and sham-controlled clinical trials. Despite methodological limitations and side effects requiring attention, electroconvulsive therapy was considered a safe and effective treatment of patients with severe agitation and related behavioral disorders due to dementia.

## RESUMO

Distúrbios comportamentais são clinicamente relevantes em pacientes com demência, e regimes farmacológicos para mitigar esses sintomas têm proporcionado resultados limitados. Comprovadamente eficaz em diversas condições psiquiátricas, a eletroconvulsoterapia é uma estratégia potencialmente benéfica para o tratamento de pacientes com agitação grave na demência.

**Objetivos:**

Esta revisão examina as publicações sobre eficácia, segurança e tolerabilidade da eletroconvulsoterapia no tratamento de pacientes com agitação na demência.

**Métodos:**

Realizamos uma análise sistemática da eletroconvulsoterapia no tratamento de pacientes com demência e agitação grave. Os artigos foram classificados quanto ao nível de evidência com base no delineamento metodológico. Os pacientes receberam um curso agudo de eletroconvulsoterapia, frequentemente seguido de manutenção.

**Resultados:**

Foram selecionados 19 estudos (156 pacientes; 64,1% mulheres; 51–98 anos): um estudo caso-controle desenvolvido com base na análise de prontuários (nível de evidência 2); um estudo aberto (nível de evidência 3); três estudos de análise retrospectiva de prontuários (nível de evidência 4); e 14 séries/relatos de casos (nível de evidência 5). Não foram identificados ensaios clínicos randomizados e controlados com placebo (nível de evidência 1), fator que representa a principal fragilidade metodológica. No entanto, o principal achado da presente revisão consistiu na ausência de desenho metodológico baseado em ensaios clínicos randomizados e controlados com placebo. Em geral, os efeitos colaterais foram transitórios e bem tolerados. Alguns pacientes apresentaram delirium pós-ictal, descompensação cardiovascular e alterações cognitivas por períodos breves.

**Conclusões:**

No geral, os pacientes obtiveram melhora significativa na agitação. No entanto, o principal achado da presente revisão foi a ausência de delineamento metodológico baseado em ensaios clínicos randomizados e controlados com placebo. Apesar das limitações metodológicas e dos efeitos adversos, a eletroconvulsoterapia foi considerada um tratamento seguro e eficaz em pacientes com agitação grave e com outros distúrbios comportamentais clinicamente relevantes na demência.

## INTRODUCTION

According to a systematic review of epidemiology, agitation and other related behavioral disorders affect 5 to 88% of patients with dementia^1^; the prevalence is associated with cognitive decline and deterioration of daily living activities^2,3^. The incidence of these manifestations is higher among patients in moderate and severe stages of dementia^4^.

Whereas prevention of neuropsychiatric symptoms (NPS) is strongly desirable, currently managing these occurrences in the daily clinical routine is a challenging assignment. As widely recognized, non-pharmacological approaches based on psychological, occupational, and/or environmental strategies are safe and tend to provide consistent benefits without worrisome adverse effects and should therefore be implemented in clinical settings prior to pharmacological treatment^5–7^.

From another point of view, the effectiveness of current pharmacological treatments available to mitigate agitation and other psychopathological manifestations in dementia is limited and, in addition, medication side effects can endanger the safety of prescriptions^8,9^.

In this scenario, electroconvulsive therapy (ECT), a treatment already proven effective for elderly patients with serious psychiatric conditions such as suicide risk, psychotic depression, schizophrenia, persistent delusional disorders, and catatonia^10–12^, may represent a promising therapeutic strategy for disruptive agitation in dementia.

Growing evidence supports that ECT is relatively safe for elderly patients, although several adverse effects have been reported, including cognitive changes and transient confusional states, which may particularly affect more vulnerable patients^13,14^. Nevertheless, these phenomena are usually transient, being resolved in a few days^14^. The management of agitation and other behavioral disturbances in dementia using ECT began decades ago, in the early 1990s^15,16^. Since then, the procedure has deserved particular attention from clinicians in the treatment of the elderly population^14^. However, relevant methodological limitations were not overcome over time. Most publications revealed interventions based on numerically limited samples, lacked randomized placebo-control groups, or open-label designs, or were restricted to retrospective chart analyses or case reports^17^. Thereby, the approaches still reflect a methodological weakness that hinders the recognition of the ECT real effectiveness in the treatment of patients with agitation and related behavioral disorders in dementia. Likewise, developing methodological designs to define the efficacy and side effect profile of ECT in patients with dementia is highly desirable and represents a relevant challenge.

To address the above questions, the purpose of the present review was to examine publications on the efficacy, safety, and tolerability of ECT in the treatment of patients with agitation and related behavioral disorders due to dementia.

## METHODS

### Search strategy and inclusion criteria

To identify appropriate studies, we searched the PubMed database considering publications up to 2022 in English, French, Italian, Spanish and Portuguese. We carried out a systematic review based on the following terms: “ECT” OR “electroconvulsive therapy” AND “dementia” OR “Alzheimer’s disease” OR “frontotemporal dementia” OR “vascular dementia” OR “mixed dementia” OR “dementia with Lewy bodies” OR “Parkinson’s dementia” OR “unspecified dementia” AND “agitation” OR “aggression” OR “psychological and behavioral symptoms of dementia” OR “BPSD” OR “behavioral disturbances” OR “behavioral disorders” OR “behavior” OR “neuropsychiatric symptoms” OR “NPS”. Additional eligible studies were searched in the references of primary publications and relevant reviews. The selected articles met the inclusion criteria, which consisted of the diagnosis of any type of mild to severe dementia and ECT for the treatment of agitation or related behavioral disorders.

### Level of evidence

We established the level of evidence of publications according to the classification suggested by Bellomo and Bagshaw^18^. Thus, a rating of “level 1” of evidence was assigned to powered randomized controlled trials (RCT) with sham; “level 2” for under-powered RCTs; “level 3” for non-randomized observational studies; “level 4” for non-randomized studies with historical controls; and “level” 5 for case series without controls.

## RESULTS

### Study characteristics and outcomes

The search yielded a total of 1,117 references, and after analyzing the title and abstract, most were excluded since they did not meet the inclusion criteria. We identified 19 studies according to the criteria established for the objective of this review, with a total of 156 patients who received ECT. Furthermore, in one of the studies,^19^ a comparison group consisting of 71 individuals was inserted. Among the selected articles, we obtained: no RCT sham-controlled (level of evidence 1); one case-control study by chart analysis (level of evidence 2); one open-label study (level of evidence 3); three historical/retrospective chart analyses (level of evidence 4); and 14 case series/reports (level of evidence 5). Of the total number of patients, 64.1% were women, and age ranged from 51 to 98 years.

In general, patients were refractory to psychotropic medication and received an acute ECT course plus ECT maintenance for several weeks, achieving significant improvement in agitation and related behavioral disturbances.

### Case-control study

Zhang et al.^19^ conducted a retrospective chart analysis based on a case-control design, with a level of evidence 2, to examine the efficacy of ECT in treating patients with any type of mild to severe dementia (Alzheimer’s disease [AD], vascular dementia [VD], mixed dementia, and others). The study included 23 patients who received ECT and 71 patients as a comparison group. The participants aged 60 years and older, and about 70% were women. The authors reported that 73.9% of them received ECT because of a high risk for suicide or aggressive behavior, insufficient response to pharmacotherapy, or intolerable adverse effects of the drugs. The average number of ECT sessions was 6.1 (±3.0), with bitemporal stimulation; the seizure adequacy was established as greater than 25 seconds according to electroencephalographic monitoring. The results showed that 91.3% of patients entirely or partially improved with ECT. Regarding adverse effects, 47.8% exhibited mild-moderate transient occurrences, such as headache, myalgia, nausea, dyspnea, and hypertension; postictal confusion was not reported. This analysis supports that ECT represents an effective and safe treatment of patients with dementia and risk of suicide or aggressiveness.

### Open-label study

A single open-label study^20^ with a level of evidence 3, included 23 patients with a mean age of 73.8 years, with moderate and advanced dementia (AD, VD, mixed dementia, frontotemporal dementia [FTD], and others) who did not acceptably respond to psychopharmacological treatment alone. Most participants received a course of right unilateral ECT, in a series of 3 to 12 sessions. When the response was not clinically satisfactory, stimulation was transitioned to bitemporal. There was a significant decline in agitation, according to the short form of Cohen-Mansfield agitation inventory (CMAI) and neuropsychiatric inventory (NPI) scores in behavioral dyscontrol and agitation domains. However, patients experienced no benefits for mood and psychosis. In addition, with the discharge from the ECT course, antipsychotics were significantly reduced. Notwithstanding, ECT was discontinued in 2 patients due to poor response, which was defined by a recurrence of persistent agitation and episodes of aggressive behavior. The treatment was also discontinued in 3 patients in consequence of adverse events, including delirium, hypertension, atrial fibrillation, ataxia, and incontinence, despite improvement in agitation and aggressive behavior. Overall, the authors admitted that the ECT course was well tolerated by most participants.

### Chart analysis of case series

The next three studies were classified as retrospective chart analyses of case series, with a level of evidence 4.

Sutor and Rasmussen^21^ administered a course of ECT to 11 patients, aged between 59 and 98 years (mean: 81.4 years), with severe AD, who exhibited psychotic symptoms, verbal or physical aggression, and whose psychopharmacological treatment failed. Patients received from 4 to 8 sessions with bitemporal (n=8), bifrontal (n=1), and right unilateral (n=2) lead placement. Of the sample, 9 patients showed clinical improvement of neuropsychiatric symptoms and 2 did not improve. As for side effects, most patients had few adverse symptoms, tolerating ECT well, except for one patient who developed atrial fibrillation during the procedure, requiring cardiological support with cardioversion.

Likewise, another chart analysis of a case series was performed by Ujkaj et al.^22^, which consisted of 16 patients (mean age 66.6 years) with clinical diagnosis of mild to severe stages of AD (n=8), FTD (n=3), VD (n=2), and unspecified dementia (n=3). Patients underwent an average of 9 ECT sessions with a range from 2 to 15, lasting approximately 90 days, after receiving psychotropic medications without adequate response. In the first course, patients received right unilateral treatment. If patients reached an insufficient response, ECT was extended to bilateral placement to maximize clinical outcomes; this strategy was applied to most participants. Behavioral disturbances such as aggressiveness, resistance to care, and especially agitation, were significantly improved. Despite post-ECT confusion in 10 patients, this occurrence was transient, and the authors considered the treatment well tolerated by most participants.

Hermida et al.^14^ developed a chart analysis involving a larger sample, consisting of 60 patients with a mean age of 77.5 years, most of them coming from assisted living facilities. Patients were clinically diagnosed with AD (n=28), VD (n=2), mixed dementia (n=6), FTD (n=2), and unspecified dementia (n=22) at moderate to severe stages, exhibiting clinically relevant behavior disorders such as agitation and aggression. Most patients did not respond sufficiently to multiple psychotropic therapies. The participants received 4 to 6 sessions of right unilateral ECT while inpatients. For 7 patients, the treatment was extended to 12 procedures or more as maintenance therapy after discharge. In 8 patients, electrode placement was changed to bifrontal due to poor response to right unilateral treatment. After discharge, no further data were assessed. Most patients got better in agitation and aggression, as well as in global daily activities. This achievement enabled the reduction of the regular use of psychotropic polypharmacy. Nevertheless, two patients were readmitted to assisted living facilities because of remaining behavioral disturbances; one of them was treated with another acute ECT course within one year but without a review of the results. Moreover, one patient presented delirium and six other patients had postictal agitation and mild confusion after the first intervention. To avoid the recurrence of these events, olanzapine 5 mg was prescribed for these patients before the subsequent ECT procedure. Hermida et al.^14^ admitted that such postictal side effects were relatively common, albeit transient, and advocated ECT as a relatively well-tolerated treatment.

### Case reports

Fourteen case reports were identified, with a level of evidence 5, each with 1 to 4 patients with severe dementia, and aged between 51 and 98 years. The most prevalent diagnosis was AD, followed by FTD. Other diagnoses were FTD overlapping with bipolar disorder, language dementia, and unspecified dementia. Patients underwent ECT since they did not respond sufficiently to psychopharmacological treatment. In 9 reports the ECT leads were placed bilaterally — bifrontal or bitemporal^11,15,16,23–29^ — followed by right unilateral ECT in 2 reports^30,31^. Two publications did not disclose the lead placement^32,33^. Noteworthy, in the report by McDonald and Thompson^11^, an 85-year-old patient had the baseline diagnosis of dementia changed to ‘pseudodementia’ because of significant cognitive improvement after ECT treatment.

Overall, the publications analyzed in this review — assembling a case-control and an open-label study, three retrospective studies based on chart analyses, and several case reports – stated relevant clinical benefits of ECT for the treatment of patients with dementia and concomitant behavioral disorders, particularly, agitation and aggressiveness. One patient with FTD and co-occurring catatonia and sexual disinhibition did not benefit from the ECT regimen, so the treatment was discontinued.

Concerning adverse effects of ECT, a postictal confusional state was the most frequent occurrence, although being a transient event. Two patients presented with clinically relevant side effects, such as cardiac instability associated with atrial fibrillation; one developed delirium, and another presented ST-segment depression, which was resolved with specific pharmacological support.

A considerable part of these publications did not include cognitive outcomes as a target of ECT treatment. Nevertheless, some of them mentioned transient memory changes, and others pointed out a stable cognitive profile or even improvement in some patients.

In summary, the main finding of the study was the lack of investigations with consistent methodological design.

Table 1 summarizes the general features from selected publications including demographic data, diagnoses of dementia, neuropsychiatric symptoms, ECT details, outcomes, as well as adverse effects and impact on cognition.

**Table 1:** Data from nineteen publications on electroconvulsive therapy for treating agitation ad related behaviors in dementia (no sham-controlled clinical trials - level of evidence 1 - were identified).

Characteristics of the 19 selected studies								
Authors	Sample (n), age (years), gender		Dementia and staging	ECT therapeutic scheme	Symptoms assessment	Outcome/efficacy	Safety and tolerability	Cognition
**Case-control study by chart analysis – level of evidence 2**								
	Zhang et al.^19^	ECT: n=23 (age: 60y and older; M: 30.4%); Comparison group n=71 (age: 60y and older; M: 31%)	Mild to severe dementia (AD, VD, mixed/AD+VD, other types) with agitation, aggression and suicide risk	Bitemporal; mean sessions: 6.1 (±3.0); seizures greater than 25 seconds; pulse width: 0.25 and 1.0 ms	CGI	91.3% fully or partially responded to ECT behavioral disorders and risk of suicide	47.8% experienced mild-moderate transient headache, myalgia, nausea, dyspnea, hypertension; no postictal confusion reported	Transient memory impairment; no persistent memory changes
**Open-label and naturalistic study – level of evidence 3**								
	Acharya et al.^20^	n=23 age: 73.8 y F: 14 M: 9	Moderate to severe AD, VD, mixed/AD+VD, FTD, unspecified dementia	Right unilateral (n=17), bilateral (n=4) transitioned to bitemporal (n=2) due to no response; 12 sessions; seizures duration not mentioned; pulse width: 0.5–1.0 ms	CMAI-short form, NPI, CSDD, CGI	Significant improvement in behavioral dyscontrol and agitation; no changes in mood and psychosis domains of the NPI	Well tolerated by most participants; 3 patients presented delirium; 1 patient developed atrial fibrillation; 1 patient presented visual hallucinations	From before to after ECT, the MMSE increased by approximately 2 points on average
**Retrospective and systematic analysis of chart review of case series – level of evidence 4)**								
	Sutor and Rasmussen^21^	n=11 Age: 81.4y (59-98y) F: 10 M: 1	Severe AD with psychosis, physical and verbal aggression; 8 patients had history of primary psychiatric disorders (depression, generalized anxiety, BP)	Bitemporal (n=8), bifrontal (n=1), right unilateral (n=2); 4–8 sessions, duration of seizures unspecified dementia	Objective behavior assessment not reported	Improvement for 9 patients; no benefits in 2 patients	Most patients tolerated the ECT well; one patient developed atrial fibrillation	Decreased cognition in 2 patients, no information for the other 9 patients
	Ujkaj et al.^22^	n=16 age: 66.6y F: 15 M: 1 (retrospective systematic chart review)	Mild to severe AD (n=8); FTD (n=3), VD (n=2); unspecified dementia (n=3) with aggression and agitation	9 sessions (from 2 to 15); bilateral (n=12), right unilateral (n=4), transitioned to bilateral when insufficient response (n=4); 9 patients received ETC maintenance; seizure duration not reported.	PAS, CGI	Significant improvement in aberrant vocalizations, motor agitation, aggressiveness, and care resistance	In general, well tolerated; 10 patients developed postictal transient confusion.	Cognition not reported after ECT course
	Hermida et al.^14^	n=60 age: 77.5y F: 45 M: 15 (case series from a chart review)	Moderate to severe AD n=28), VD (n=2), FTD (n=2), mixed (n=6), unspecified dementia (n=22) with aggression and agitation.	Right unilateral; 8 patients transitioned to bifrontal due to poor response; 4−12 sessions (average: 9.6); seizure duration not mentioned; width pulse: 0.37 ms	PAS, GAF, psychiatric consult notes	Improvement in global functioning with decrease of psychotropic polypharmacy	Postictal agitationand mild postictal confusion in 6 patients; delirium in 1 patient	Cognition not reported after ECT course
**Case reports – level of evidence 5**								
	Carlyle et al.^15^	n=3 Age and gender not reported	Unspecified dementia characterized by substantial cognitive decline and agitation	Bilateral; number of sessions and seizure duration not mentioned	Objective behavior assessment not reported	Rapid resolution of screaming	Tolerability or side effects not reported	Cognition not reported after ECT course
	Holmberg et al.^16^	n=1 age: 78y, F	Severe VD	Bilateral; seizure duration from 45 to 60 seconds; 54 sessions; maintenance for 2 years; brief-pulse width	Behavioral rating scale	Dramatic improvement in agitation and aggression at the beginning of treatment; maintenance of ECT for 2 years with response oscillation	Well tolerated	Cognition not reported after ECT course
	Roccaforte et al.^32^	n=1 age: 77y, F	Language advanced dementia; disruptive vocalizations, verbal aggression, warbling, loudly, obscenely	6 sessions; lead placement not reported; seizure duration not reported	Objective behavior assessment not reported	Substantial improvement	Tolerability or side effects not reported	Before ECT the MMSE was 0, but not assessed after the procedure course
	Grant et al.^23^	n=4 Case 1: age: 56y, F	Severe pre-senile AD with agitation and suspected visual hallucinations	Bilateral, 4 sessions; seizures lasting 37–43 seconds; brief-pulse width	Objective behavior assessment not reported	Improvement in agitation and aggression	Postictal confusion lasting several hours after treatment	In the 4 cases, since dementia was severe, the risk of cognitive worsening was considered moot
		Case 2: age: 78y, M	Severe AD with agitation and aggression	Bilateral; 7 sessions; seizures lasting 26–39 seconds; brief-pulse width	Objective behavior assessment not reported	Dramatically reduction of agitation and aggression	Tolerability or side effects not reported	
		Case 3: age: 77y, F	Severe AD with agitation, sleep disorder	Bilateral; 2 sessions; seizures with 46 seconds and 52 seconds; brief-pulse width	Objective behavior assessment not reported	Improvement in agitation, verbal aggression, and sleeping	Tolerability or side effects not reported	
		Case 4: age: 78y, F	Severe AD with agitation and verbal aggression	Bilateral; 4 sessions in the first period and 4 additional sessions after three months (8 sessions); seizures lasting 30–54 seconds; brief-pulse width	Objective behavior assessment not reported	Improvement in verbal aggression and repetitive speaking after first course; no additional information	Tolerability or side effects not reported	
	McDonald and Thompson^11^	n=3 Case 1 Age: 73y M	Severe Lewy-body dementia and BP, with mania symptoms, agitation, and aggressiveness	Right-unilateral acute course; 8 sessions plus maintenance ECT every 2 weeks for the following 18 months; seizures duration not mentioned; 8 brief-pulse width	Objective behavior assessment not reported	Marked improvement in mood and behavior	No postictal confusion or worsening in dementia	Cognition improved – MMSE from unobtainable to 9 out of 30
		Case 2 Age: 85y F	Moderate dementia not otherwise specified with agitation and aggressiveness and mania symptoms; history of alcohol and benzodiazepines dependence	Right-unilateral ECT acute course; 6 sessions with maintenance course for 6 months; seizures duration not mentioned; 6 brief-pulse width	Objective behavior assessment not reported	Significant improvement in mood and behavior	No postictal confusion or worsening in dementia	Cognition remained stable; MMSE 15–16 out of 30
		Case 3 Age: 71y F	Mild-moderate dementia not otherwise specified and BP with mania symptoms and agitation	Right-unilateral ECT acute course; 7 sessions with maintenance course for the subsequent 18 months; seizures duration not mentioned; 7 brief-pulse width	Objective behavior assessment not reported	Complete resolution of mania and agitation	No postictal confusion or worsening in dementia	Cognition improved; MMSE from 24 to 28 out of 30. Later this patient was diagnosed with “pseudodementia”
	Bang et al.^24^	n=2 Case 1: age: 88y, F	Severe AD+PDD with verbal agitation, obscenities, hollering, palilalia, and screaming;	Bilateral; 11 sessions; seizures up to 84 seconds	Objective behavior assessment not reported	Marked improvement in verbal agitation	Well tolerated	The patient improved in verbal language and in nodding in response to questions
		Case 2: age 80y, F	Severe unspecified dementia, with agitation, screaming, spitting, striking	Bilateral; 5 sessions; seizures up to 56 seconds	Objective behavior assessment not reported	Improvement in verbal agitation and aggression	Well tolerated	Cognition not reported after ECT course
	Suzuki et al.^25^	n=1 age: 51y, M	FTD overlapping severe BP with angering, joking, followed by apathy or agitation	Bitemporal; 9 sessions; seizures lasting 39–70 seconds; brief-pulse width	Objective behavior assessment not reported	Improvement in behavioral disturbances	High fever, liver dysfunction and ECT interruption for 1 week, being resumed afterward	Improvement in attention and abstract thinking
	Wu et al.^26^	n=2 Case 1: age: 78y, M	Severe AD with aggression	Bitemporal; 7 sessions, seizures lasted up to 65 seconds	Objective behavior assessment not reported	Marked improvement	Well tolerated	Cognition not reported after ECT course
		Case 2: age: 71y, M	Severe FTD with pushing, grabbing, choking	Bitemporal; 6 sessions, seizures lasted up to 58 seconds; maintenance for every 28 days	Objective behavior assessment not reported	Improvements in aggression	Well tolerated	Cognition not reported after ECT course
	Paul et al.^33^	n=1 Age: 51y, M	FTD with history of bipolar disorder; unreported severity	9 sessions; localization not reported; seizures duration not reported	Objective behavior assessment not reported	Significant improvement in mood symptoms without benefits for erratic behavior and laughter	Tolerability or side effects not reported	The MMSE score went from 6/28 before ECT up to 20/28 after treatment (two items were excluded: the drawing and written portions of the exam)
	Aksay et al.^30^	n=1 Age: 57y, F, right-handed	Severe pre-senile AD with agitation, aggressiveness, and repetitive outbursts	Right unilateral, 8 sessions; seizures duration not mentioned; pulse width: 0.25 ms	PAS	Significant improvement	Well tolerated despite headache; 3 spontaneous self-limiting generalized seizures.	ECT without any recognizable worsening of cognition
	Borisovskaya et al.^31^	n=1 age: 70y, M	Moderate to severe FTD with perseverative behavior, psychosis, apathy, and depression	Right unilateral, 6 sessions; seizures average of 60 seconds; several months with maintenance treatment; ultrabrief pulse of 0.3 ms	FAB	Initial improvement and subsequent worsening	ST-segment depression at EKG.	No worsening in cognition
	Fazzari et al.^27^	n=1 age: 76y, F	Severe AD with agitation, insomnia, mood lability, weeping, anger, repetitive vocalization, and swearing	Bitemporal and bifrontal: 21 sessions including acute course and maintenance; seizure duration not mentioned.	Objective behavior assessment not reported	Resolution of confusional state, psychomotor agitation and behavioral disturbance	Tolerability or side effects not reported	Cognition not reported after ECT course
	Selvadurai et al.^28^	n=1 age: 64y, M	Severe unspecified dementia, with agitation and aggression	Bitemporal with 15 sessions for an acute course followed by a weekly and until every 5 weeks for maintenance was continued by a 17 month-period; seizure duration not reported	NPI-C	Substantial and sustained improvement of agitation and aggression	Discontinuation of the ECT acute phase due to pneumonia; later ECT was resumed	Prior ECT cognition assessment was not achievable due to aggressiveness; however, ECT was beneficial for cognition
	França et al.^29^	n=1 age: 95y, F	Severe late-onset FTD with sexual disinhibition, aggressiveness, agitation, bizarre social behavior, followed by catatonic stupor	Bitemporal; 6 sessions; seizures lasting 20–30 seconds; brief-pulse width	Bush-Francis Catatonia Rating Scale; Northoff Catatonia Scale	No benefits, and interruption of ECT course	Tolerability or side effects not reported	Cognition not reported after ECT course

Abbreviations: AD: Alzheimer’s disease; BP: bipolar disorder; CGI: clinical global impression; CMAI: Cohen-Mansfield agitation inventory (short form); CSDD: Cornell scale for depression in dementia; PDD: Parkinson’s disease dementia; ECT: electroconvulsive therapy; EKG: electrocardiogram; F: female; FAB: frontal assessment battery; FTD: frontotemporal dementia; GAF: global assessment of functioning; M: male; MMSE: mini-mental state examination; MND: major neurocognitive disorder; NPI: neuropsychiatric inventory; NPI-C: neuropsychiatric inventory-clinician; PAS: Pittsburgh agitation scale; VD: vascular dementia.

## DISCUSSION

Based on this systematic review, we detected a significant improvement in agitation and related behavioral disorders in patients with severe forms of dementia due to AD, FTD, VD and non-specified etiologies. Patients underwent ECT treatment when psychopharmacological regimens failed to control neuropsychiatric symptoms, or if they exhibited relevant medication side effects.

Due to the variability in the number of patients and diagnoses of dementia, the different tasks to assess neuropsychiatric symptoms, as well as the dissimilar number of ECT sessions and electrical charges in each intervention, it is not achievable to produce a meta-analysis of selected publications. The availability of data in this matter is restricted to those reported by 19 selected publications: one retrospective case-control study, one open-label study, three retrospective chart analyses, and 14 case reports. In spite of noticeable efforts to improve the methodological design over decades^17^, we acknowledged the lack of randomized, placebo-controlled clinical trials addressing the safety and efficacy of ECT for the treatment of patients with severe agitation in advanced stages of dementia. We understand that this is a significant gap in the medical literature, given that ECT, on clinical grounds, has been recommended for severe psychological and behavioral symptoms of dementia, unresponsive to conventional pharmacotherapy and other non-pharmacological interventions.

Despite such limitations, in a retrospective case-control study, Zhang et al.^19^ described clinically favorable responses to ECT for the treatment of patients with dementia and risk of suicide and aggression, with relatively well tolerability. Likewise, in an open-label study, Acharya et al.^20^ found a marked betterment in agitation, behavior dyscontrol, and aggression in patients with moderate to severe dementia after the ECT regimen, with subsequent reduction in antipsychotic prescriptions.

Overall, according to this review, ECT substantially improved severe agitation and related behavioral disturbances in patients with different types of dementia (AD, FTD, VD, Parkinson’s disease dementia [PDD], language dementia). Although some studies showed a significant number of patients who required maintenance of ECT for an extended period after discharge from the first course, the intervention brought relevant clinical benefits for this group. Noteworthy, another relevant outcome reported by several studies was the reduction in the number of routine prescriptions for psychotropic drugs.

A point that deserves special attention concerns the grouping of individuals with different types of dementia within the same analysis. Unlike case reports of a single patient, in which the diagnosis was usually described, in the other studies this definition was not clear. These studies grouped subjects with different types of dementia, thereby making it impossible to identify the efficacy of the ECT, as well as adverse effects, for patients according to the specific diagnosis. Furthermore, large variations regarding the number of patients, number of sessions, seizure lasting, lead position (unilateral, bitemporal, or bifrontal), as well as the amount of electrical charge applied were also quite dissimilar. Together, such circumstances represent a caveat for extending the results to patients with a specific diagnosis of dementia.

Regarding safety, clinically relevant adverse effects were restricted to postictal delirium, transient mental confusion, and atrial fibrillation. In the study carried out by Acharya et al.^20^ with 23 subjects, 2 patients were discontinued because of delirium. Moreover, 10 patients in the chart analysis performed by Ujkaj et al.^22^ had a postictal confusional state, and the authors assumed that the episodes were usually transient and did not meet the diagnostic and statistical manual of mental disorders (DSM-5) criteria for delirium. Furthermore, Hermida et al.^14^ reported postictal agitation in 6 patients from a larger sample (n=60) in the context of mild confusion, mainly during the first treatment; to prevent similar events, olanzapine 5 mg was prescribed for them before subsequent ECT procedures.

Postictal delirium has been consistently reported in elderly patients. In a systematic review of 488 individuals undergoing ECT, Grover et al.^34^ detected prolonged postictal delirium in 5.7% of participants aged 42 years and older, with depression or affective disorders. According to Grover et al.^34^ review, delirium was attributed to higher doses of quetiapine and lack of antidepressants during the ECT course, without any other substantial related factors. Conversely, as mentioned above, olanzapine, a sedative antipsychotic drug, was prescribed to prevent postictal mild confusion and delirium^14^. Furthermore, in a retrospective chart review on ECT to treat patients up to 82 years of age with major depression and other psychiatric diagnoses, Jo et al.^35^ found 8.6% of 268 sessions associated with postictal delirium, *w*hereas another review reported that ECT was a safe treatment^36^. As the aforementioned adverse effects were detected in patients with different diagnoses of dementia, comparing data from these approaches with our results seems inconsistent.

Presumed factors acting synergistically can induce brain disorganization and mental confusion. Predisposing conditions are dementia *per se*, primarily with impaired cholinergic activity, cerebrovascular disease affecting subcortical brain nuclei, prolonged ECT-related seizures, and medications with potential adverse impact on brain functioning, among others^34,37^. An intriguing matter about the risk for delirium concerns ECT dosage and seizure threshold. Selvaraj and Prahara^37^ argued that a higher stimulus dosage could increase the severity of postictal delirium. Besides, the magnitude by which the electrical charge exceeded the appropriate seizure threshold contributed to that occurrence^38^.

Another issue refers to the potential cardiac changes of ECT in the elderly, as they are clinically more susceptible to adverse effects. Thus, among 11 patients enrolled in Sutor and Rasmussen^21^ chart analysis, one patient developed atrial fibrillation during the ECT course, so the treatment was discontinued; he resumed the procedure after the resolution of the heart disorder by cardioversion. Another patient in the Acharya et al.^20^ study also developed atrial fibrillation requiring specific pharmacological support. In addition, Borisovskaya et al.^31^ described ST-segment depression observed on the electrocardiogram, with interruption of the ECT course; it was restarted as the patient recovered from his previous cardiological condition. In this scenario, cardiac events related to ECT should be carefully monitored as the elderly are particularly vulnerable to procedures that demand sedation or anesthesia.

Cognitive changes were recurrent complaints in old people undergoing ECT, including anterograde memory loss. Although usually reversible within weeks, such occurrences were of more concern in patients with dementia due to their greater vulnerability to side effects^13,14^. Even in elderly inpatients with major depressive disorder having concomitant mild cognitive impairment or dementia undergoing ECT, cognitive changes were transient^39^. The impact of ECT on cognition may be associated with coexisting presumptive factors, for instance, stage of dementia, cerebrovascular disease, concomitant medicines, and unilateral or bilateral lead placement, among others.

Unsurprisingly, a formal assessment of cognition was not performed before the ECT regimen because of severe agitation, making it difficult to achieve its real impact on postictal cognitive status. Despite these methodological disadvantages, some studies were able to assess a slight improvement or stability of patients’ cognition, and they attributed such benefits to the ECT efficacy in controlling agitation or behavioral disorders^11,20,24,25,28,30–32^. In this context, decreased cognition after the ECT course was confirmed only by Sutor and Rasmussen^21^ in 2 patients out of a total of 11 participants. Furthermore, in Grant an Mohan^23^ study, the worsening of cognition was debatable and could be attributed to the severe stage of dementia *per se*. Nevertheless, in most studies, cognition was not reported, and due to agitation and behavioral disturbances, among others, a standardized cognitive assessment was not available^14–16,22,24,26,27,29,32^.

It is conceivable that elderly patients with a cholinergic signaling deficit may be at increased risk of post-ictal cognitive changes, including memory impairment and delirium related to the ECT procedure^40^. Although the ECT intervention initially induces an ictal peak of acetylcholine, later, in the post-procedure phase, a decrease in cholinergic activity was detected^40^. Thus, patients with dementia characterized by marked hypocholinergic activity, for instance AD, who are particularly vulnerable to delirium or cognitive impairment, deserve special attention in the treatment with ECT.

As it is known, in FTD and other non-marked hypocholinergic dementias, the aforementioned side effects can reflect different neurotransmitter systems other than acetylcholine, including serotonin, dopamine, gamma-aminobutyric acid (GABA) and, most of all, glutamate^41^. The presumed role of such compounds in the ECT intervention for the management of behavioral disturbances in patients with AD and other types of dementia can be conjectured, and further investigations are highly worthwhile.

Concerning the risk of mortality associated with ECT, epidemiological studies involving the elderly are scarce. Based on an analysis of data extracted from medical records over 17 years on ECT for primary psychiatric conditions, Liang et al.^42^ noticed a non-increased risk of mortality among patients who were treated, with a rate of 1.27%, compared to 1.94% of those who did not receive the intervention. Nevertheless, the authors warn that the patients, in general, were adults and without relevant medical comorbidities, a common phenomenon in the elderly. In turn, Nuttall et al.^43^ also performed a retrospective analysis of medical records of patients with primary psychiatric conditions, with a mean age of 63 years, and did not find an increase in the mortality rate as a result of ECT. However, the most clinically relevant adverse effects, i.e., respiratory events, occurred among the elderly. Thus, the risk of relevant adverse effects on the elderly deserves special attention when prescribing ECT; this care will certainly contribute to reducing the prejudices related to this therapeutic practice still existing in the community.

Despite the occurrence of postictal delirium, behavioral confusion, cardiological events, or cognitive changes in some patients, in general, adverse side effects were transient, and ECT was well tolerated.

Although the number of participants was generally small, diagnoses varied substantially: AD, followed by FTD, FTD overlapping bipolar disorder, VD, dementia with depression, PDD, and unspecified dementia. Some participants presented early-onset dementia, between 51 and 64 years old, and were classified as having pre-senile dementia when they underwent ECT treatment, while others were very old, reaching 95 or 98 years of age at the time of the intervention.

For the ECT procedure, most of them were applied in bilateral placements (bifrontal or bitemporal) followed by right unilateral location. In three studies the lead placement was transitioned from right unilateral to bilateral to achieve more consistent efficacy^14,20,22^. During the acute phase, there was no report on change in the stimulus location from bilateral to unilateral. Besides, two studies did not disclose the position of the electrodes^32,33^. Probably due to the severity of agitation and other behavior disorders, the choice of stimulus application was predominantly bilateral. Most patients included in case reports or case series received bilateral stimulation followed by right unilateral. In the Ujkaj et al.^22^ study, as the results were not convincing for 12 patients out of a total of 16, the procedure was switched from unilateral to bilateral location to maximize clinical response. With this arrangement, patients in general reached significant clinical improvement.

The main finding of the present review reflects the methodological weaknesses of the studies attributed to the absence of randomized clinical trials, placebo-controlled, and with suitable samples. Such limitation was also reported previously^17^. Notably, different diagnoses of dementia were considered within the same analysis, including AD, FTD, DV, or unspecified dementia. In addition, each dementia requires different routine drugs concomitant with ECT treatment, also interfering with the behavioral profile. Together, these factors make it difficult to generalize the results of studies. Additional investigations should address ECT treatment of agitation and other neuropsychiatric symptoms with reliable methodological design.
